# Strategies to Promote Long-Term Cardiac Implant Site Health

**DOI:** 10.7759/cureus.12457

**Published:** 2021-01-03

**Authors:** Jane Taleski, Biljana Zafirovska

**Affiliations:** 1 Electrophysiology and Electrostimulation, University Clinic of Cardiology, Skopje, MKD; 2 Interventional Cardiology, University Clinic of Cardiology, Skopje, MKD

**Keywords:** cardiac implantable electronic device (cied), central venous access, incision site, wound suture, pacemaker complications

## Abstract

In the past several decades there has been a continuous growth in the field of cardiac implantable electronic devices (CIED) implantation procedures as well as their technological development. CIEDs utilize transvenous leads that are introduced into the heart via the axillary, subclavian, or cephalic veins, as well as a devices generator that is implanted in a subcutaneous pocket, typically in the pre-pectoral region. Despite this significant improvement, complication rates range from 1-6% with current implant tools and techniques. In this review we will discuss the three central parts of the CIED implantation procedure, their impact on implantation site, infections, and possibilities for its prevention.

## Introduction and background

Pacemakers (PM), implantable cardiac defibrillators (ICDs), and cardiac resynchronization therapy (CRT) devices are life-saving treatments for many cardiac conditions [1]. Since the introduction of cardiac implantable electronic devices (CIED), there had been a continuously expanding set of indications for their use in patients with conduction abnormalities and cardiomyopathies, together with steadily increased implantation rates and technological advancement [2,3]. The approach to cardiac PM and ICD implantation has evolved during the past decades and the introduction of CRT has resulted in an increased level of complexity of the procedures [4]. CIEDs utilize transvenous leads that are introduced into the heart via the axillary, subclavian, or cephalic veins, as well as a devices generator that is implanted in a subcutaneous pocket, typically in the pre-pectoral region. Despite this significant improvement in the process of implantation, complications can occur with either component of the device/lead being implanted or explanted [5]. Complication rates range from <1% to 6% with current implant tools and techniques. These are widely divided into immediate/procedure-related, intermediate-term, and long-term or later developed complications [6].

## Review

In this review, we will discuss the differences between different currently used CIED implantation approaches and techniques. In order for better understanding, we focused our attention on three specific parts regarding the CIEDs implantation process, which is associated with the majority of the procedural complications and hopefully can help in their prevention.

Incision sites

CIED implantations are most often performed in the left sub-pectoral region. During this procedure a small 2.5-3.5cm incision is made. There are two most commonly used incision sites during CIED implantation procedures. The first type of incision is inferior and parallel to the clavicle, placed in the triangle over the anterior chest from the shoulders to the xiphoid line; this is called “infraclavicular” or C-type incision. The second type of incision is along the deltopectoral groove; “deltopectoral” D-type incision [7]. Both incision sites have their advantages and disadvantages, regarding the central vein access (cephalic, subclavian, or axillary vein) used for lead placement and late postprocedural scarring. 

The infraclavicular or C-type incision provides access to both the cephalic vein when a cut-down technique is used and the subclavian vein (puncture technique) and it is used for either subcutaneous or subpectoral pocket formation. However, this access site can be very challenging for performing the cephalic vein cut-down and axillary vein puncture technique. In comparison, the deltopectoral D-type incision is made approximately 2 cm below the clavicle, in the deltopectoral groove (indentation between the clavicular head of the pectoralis major medially and the deltoid laterally). Because this incision type runs along the cephalic vein, it allows a much simpler way to explore the cephalic cut-down technique. This D-type incision also provides easier and unique access to the axillary vein (puncture technique). This type may limit access to the subclavian vein (puncture technique) and the pocket needs to be made medially to the incision site [8]. Anatomically, an incision along the Langer’s lines, which run perpendicular to the clavicula, should heal better and, therefore, the incision along the deltopectoral groove may be considered more natural and superior to the incision parallel to the clavicle. Hence the D-type incision runs parallel to the Langer’s lines in the deltopectoral groove [9]. In one study two incision sites for pacemaker implantation were compared and their influence on scarring in a period of six months. The likelihood of scar scores with reduced severity in the deltopectoral groove incisions was not statistically significant as compared to the patient group which received infraclavicular incisions. Of course, mainly due to the small number of patients included, this study had its limitations. However, it concluded that there is further need for clinical trials to strengthen the position of deltopectoral groove incision D-type over the conventional infraclavicular incision method or C-type of incision [8]. In addition to electing a proper incision site, especially in patients with a previously implanted temporary PM, it is worth mentioning that the period between the temporary and the permanent PM implantation is very important for infection prevention, i.e., a shorter period is connected with lower infection rate [10].

This first part of our journey, called CIED implantation procedures and techniques, regarding the incision site, is in favor of deltopectoral D-type of incision, due to the easier access to the cephalic and axillary vein. If we agree that the cephalic vein cut-down technique and axillary vein puncture technique are two of three widely used main lead access sites, we can conclude that deltopectoral D-type incision is more favorable over the infraclavicular C-type incision. Regarding the scarring, comparing booth incision sites, deltopectoral D-type is probably preferable, but as we concluded additional clinical trials are needed to prove this point.

Central venous access

Gaining central venous access is a central part of the implantation procedure of CIEDs. However, there are no strict recommendations about the first access site choice, so in practice, this decision depends on operator preference and experience [11]. Cephalic vein (CV) cut-down and subclavian vein (SV) puncture are both widely used techniques for lead insertion during CIED implantation procedures [12]. Recently, the fluoroscopy-guided axillary vein (AV) puncture technique has been presented as an alternative approach and it seems that it is associated with higher periprocedural success and lower overall complications [11,13]. In one recent study, these previously mentioned three central venous access sites for CIED implantation were sought to evaluate their efficacy and safety [14]. The main findings of this study were first that cephalic vein cut-down technique was associated with a lower risk of pneumothorax and lead failure compared with subclavian vein puncture technique and there was no significant difference in risk of pocket hematoma/bleeding or device infection with CV cut-down compared with SV puncture technique. In addition, acute procedural success was higher with axillary vein and SV puncture technique compared with CV cut-down and the procedure time was significantly longer with CV cut-down compared with AV, but not with SV puncture technique [14]. CV cut-down technique was found to be the venous access of choice in 60% of the centers in Europe in a Europace published survey [4]. There are many clinical studies focused on exploring the CV cut-down technique and its many variations. In one study, a scoring system for cephalic vein assessment was developed and evaluated, based on a venogram performed before each venous access attempt. This scoring system was introduced to simplify the selection of appropriate patients to undergo the CV approach, possibly facilitating the performance of this technique especially for less experienced implanters and eventually increasing the efficiency and safety of the procedure [15]. In a different trial, the CV cut-down technique was compared to the fluoroscopy-guided AV puncture technique. Results showed that AV access is safe, has a better success rate and shorter procedural time compared with the cephalic vein access. When the results were compared among the trial operators, no differences were found in the AV or in the CV group regarding success rate, complication rate, or time to successful access [11]. Subclavian vein puncture technique has a faster learning curve and in general higher success rate [16]. However, due to its anatomic characteristics, complications are relatively common, including pneumothorax, hemopneumothorax, inadvertent subclavian artery puncture, brachial nerve plexus injury, subclavian crush syndrome, and electrode lead fracture [17,18]. In one randomized study, AV was compared to SV puncture technique for CIED implantation. The results showed that the axillary venous approach may be superior to the conventional subclavian vein approach for CIED lead placement due to lower complication rates and similar procedural time. However, the same trial noted that the AV puncture technique is more complex and has a longer learning curve [19]. Ultrasound-guided access for permanent pacemaker lead placement may be more effective and have less serious complications. One clinical trial focused on researching the learning curve for ultrasound-guided placement of permanent pacing leads and compared it with the standard cephalic vein cut-down technique. This technique was proven to be quicker to learn and achieved faster lead placement times with shorter and more predictable fluoroscopy time when compared with the cephalic vein cut-down technique. The same trial reported a success rate of over 87% in favor of the ultrasound-guided technique [20]. The use of ultrasound to improve axillary vein access and minimize complications during pacemaker implantation was reported on 403 consecutive patients. The rate of success was 99.25% and there were no access-related complications. The described technique has the potential to improve the success rate of axillary vein access and minimize complications during pacemaker implantation [21]. We can conclude that choosing the right vein for central venous access, and most importantly proper technique, together with appropriate learning curve, is the main goal for successful CIED implantation with fewer complications. Perhaps axillary vein puncture and cephalic vein cut-down techniques, with their relatively new addition tools, may be the new central techniques for providing central venous access.

Wound closure and antibiotic envelope experiences

With the advancement in technology and widespread accessibility to CIEDs around the world, an entity called CIED-related infections emerged. The incidence of these infections over the past decade was estimated at 1% to 4%, including all device implantations, despite the use of well-known prophylactic strategies such as pre-operative antibiotics and sterile surgical techniques, leading to significant morbidity, mortality, and enormous cost to the health care system [22]. However, we should also keep in mind that the infection rates after implantation of a new device are reported to be around 1%, whereas infection rates after device generator replacements are significantly higher with a reported average of up to 5% per year [23-26]. Infections can have a devastating effect on the patient, leading to possible sepsis and almost always to complete removal of the CIED system [23-27]. It has been estimated that around 50% of CIED infections are related to two gram-positive bacteria, *Staphylococcus aureus* and *Staphylococcus epidermidis*, and it is also believed that the whole process stems from contamination of the subcutaneous pocket that harbors the CIED generator [28,29]. To help minimize this possible complication after CIED implantation, which is connected with the wound, one study developed a modified wound closure technique. It was designed by a plastic surgeon and performed by an electrophysiologist. After a brief learning curve, the closure technique could be quickly and easily performed within 10 minutes after closure of the deep fascial layer. One of the benefits of this technique is that the patient was able to shower immediately after the procedure in contrast to the traditional closure techniques that delays showering for up to one week after the procedure [30]. It is worth mentioning that the technique described in this trial is probably frequently used by plastic surgeons and has only two layers of suture instead of four, five, or more that are widely used by CIED implanters.

Over the years CIED implanters have explored different suture techniques in order to minimize pocket infections, and the results suggest that suture technique does not alter PM generator pocket infection rate. Also, pocket toileting by antibiotics does not have a role in PM pocket infection rate. According to European Heart Rhythm Association (EHRA) recommendations, pocket hemostasis is the most important factor for prevention of PM pocket infection as well as proper surgical asepsis practice and the use of preoperative antibiotics [1].

To address these concerns, and with the aim of reducing CIED related infections, a multifilament mesh envelope that elutes two antibiotics, rifampin and minocycline, was approved by the Food and Drug Administration (FDA) in 2008 for CIED stabilization [31]. To efficiently utilize the antibiotic envelope in clinical practice, Mittal et al. conducted a regression model to identify independent risk factors for CIED infections. In this retrospective study, 2,891 patients were included who underwent a CIED implantation procedure and seven independent risk factors for CIED infection were identified: male sex, heart failure, hypertension, glomerular filtration rate <60 mL/min, diabetes mellitus, early pocket re-exploration, and undergoing a device upgrade. Risk score was constructed that ranged from 0 to 25, dividing the patients into those with low risk (0-7) with 1% infection risk; medium risk (8-14) with 3.4% infection risk; and high risk (≥15) with >11% infection risk. They were also able to conclude that an envelope was unnecessary when implanting a permanent pacemaker (PPM) regardless of risk group and in one-third of implantable cardioverter defibrillators or cardiac resynchronization therapy-defibrillator (ICD)/(CRT-D) procedures in the low risk (score 0-7) group [32]. The WRAP-IT trial is the only randomized controlled trial and the largest study to date that utilized antibiotic envelopes intending to reduce major device-related infections. In 6,983 patients with an increased risk for CIED-related infections, including patients undergoing generator replacements, device upgrade or revisions, and those undergoing an initial CRT-D, 3,495 patients were randomized to the envelope group. Based on results from the WRAP-IT trial there was nearly a 50% reduction in risk of CIED infection in patients that received an envelope undergoing a high power device procedure (ICD or CRT-D) compared to control, however, there was an equal amount of CIED infections in both groups of patients undergoing implantation of a lower power device (PPM or CRT-P), which is similar with the conclusion made by the Mittal et al. trial [33]. One recent meta-analysis demonstrated several important findings regarding the use of an antibiotic envelope to reduce major CIED-related infections. Collectively, the risk of major CIED-related infection was significantly reduced by 66% in patients receiving an antibiotic envelope when compared to control. In a subgroup analysis including studies that exclusively enrolled patients at high risk for CIED-related infections, the use of an antibiotic envelope was associated with a 74% reduction in major CIED-related infections. Additionally, to derive the greatest benefit of the antibiotic envelope, the decision to use it at the time of CIED implantation should be individualized based on the predetermined risk for infection in each patient [22]. With all this said, it’s necessary to mention the need for more clinical trials in this area so we can have better information about CIED infection-related complications and their most favorable prevention techniques and treatment. 

## Conclusions

Incision site, central venous access, and wound closure are only parts of the big puzzle of CIED implantation procedures and techniques. The incision site is probably, like every other part of this brief review, associated with the strategies of the selected device implantation center, learning process, and experiences. Incision type D is more efficient over the infraclavicular C-type, when cephalic vein cut-down or axillary vein puncture is the targeted technique for gaining central venous access. This incision site is also connected with less local scarring. Various centers have different strategies regarding the central access vein for CIED lead placement. It is definite that in the majority of cases, the cephalic vein cut-down technique is getting less attention, probably because it's often more time-consuming and has a longer, more difficult learning curve. Subclavian vein puncture technique is widely used, considering it is easy to perform especially with several useful tips and tricks like preprocedural/procedural contrast-enhanced venogram, anatomical markers, ultrasound-guided, and many others. But due to the most common complications connected with this puncture technique, axillary vein puncture is getting its rightful place in the process of choosing the right technique for central venous access. In addition to wound closure, the use of an antibiotic envelope in selective patients can be very helpful in prevention of CIED implantation infections, especially in high-risk patients. Hopefully this focused review will provide an additional summary of evidence and data to reduce the percentage of infections from these procedures.
